# Neurofunctional Segmentation Shifts in the Hippocampus

**DOI:** 10.3389/fnhum.2021.729836

**Published:** 2021-11-01

**Authors:** Jennifer L. Robinson, Xinyu Zhou, Ryan T. Bird, Mackenzie J. Leavitt, Steven J. Nichols, Sara K. Blaine, Gopikrishna Deshpande

**Affiliations:** ^1^Department of Psychological Sciences, Auburn University, Auburn, AL, United States; ^2^Department of Electrical and Computer Engineering, Auburn University Magnetic Resonance Imaging Research Center, Auburn University, Auburn, AL, United States; ^3^Center for Neuroscience Initiative, Auburn University, Auburn, AL, United States; ^4^Alabama Advanced Imaging Consortium, Birmingham, AL, United States; ^5^Quora Inc., Mountain View, CA, United States; ^6^Key Lab for Learning and Cognition, School of Psychology, Capital Normal University, Beijing, China; ^7^Department of Psychiatry, National Institute of Mental Health and Neurosciences, Bengaluru, India; ^8^Center for Brain Research, Indian Institute of Science, Bengaluru, India

**Keywords:** clustering, fMRI, 7T, topography, memory, connectivity, hierarchical clustering

## Abstract

The hippocampus is one of the most phylogenetically preserved structures in the mammalian brain. Engaged in a host of diverse cognitive processes, there has been increasing interest in understanding how the hippocampus dynamically supports these functions. One of the lingering questions is how to reconcile the seemingly disparate cytoarchitectonic organization, which favors a dorsal-ventral layering, with the neurofunctional topography, which has strong support for longitudinal axis (anterior-posterior) and medial-lateral orientation. More recently, meta-analytically driven (e.g., big data) approaches have been employed, however, the question remains whether they are sensitive to important task-specific features such as context, cognitive processes recruited, or the type of stimulus being presented. Here, we used hierarchical clustering on functional magnetic resonance imaging (fMRI) data acquired from healthy individuals at 7T using a battery of tasks that engage the hippocampus to determine whether stimulus or task features influence cluster profiles in the left and right hippocampus. Our data suggest that resting state clustering appears to favor the cytoarchitectonic organization, while task-based clustering favors the neurofunctional clustering. Furthermore, encoding tasks were more sensitive to stimulus type than were recognition tasks. Interestingly, a face-name paired associate task had nearly identical clustering profiles for both the encoding and recognition conditions of the task, which were qualitatively morphometrically different than simple encoding of words or faces. Finally, corroborating previous research, the left hippocampus had more stable cluster profiles compared to the right hippocampus. Together, our data suggest that task-based and resting state cluster profiles are different and may account for the disparity or inconsistency in results across studies.

## Introduction

Arguably one of the most preserved neural structures across species, the hippocampus has been a prime target for evolution theorists and cognitive neuroscientists alike. Remarkably, functional differentiation within the hippocampal formation has been posited in nearly all species over the past half century, with theories ranging from hemispheric specialization to more intricate models of topographical/subfield specialization. Support for these hypothesized compartmentalizations, especially in the human functional neuroimaging literature, has been limited in scope due to the methodological approaches employed which have largely been either lesion (e.g., case) studies, or meta-analytically driven (e.g., big data) (Robinson et al., 2015; Plachti et al., 2019). The former is hindered by generalizability concerns, while the latter lacks granularity and may not be sensitive to important task-specific features such as context, cognitive processes recruited, or the type of stimulus being presented. As such, an important gap in the literature remains as to whether these functional parcellations are stable, or if they may shift depending on such task/stimulus features contributing to some of the variability seen across studies.

Theories regarding hippocampal specialization date back to 1901 when Ramon Cajal described the cytoarchitectonic differences between hippocampal subfields (Cajal, 1901), yet the precise neurofunctional underpinnings of these anatomical differences have yet to be elucidated. Converging evidence across species suggests that the *functional* topography is generally accepted to have an anterior-posterior organization (Colombo et al., 1998; Poppenk et al., 2013; Maruszak and Thuret, 2014; Kim, 2015; Nakamura and Sauvage, 2015; Robinson et al., 2016; Hrybouski et al., 2019; Przeździk et al., 2019; Grady, 2020), in direct opposition to the known configuration of the *anatomical* subfields (which are more aligned in a dorsal-ventral fashion) (Vos de Wael et al., 2018). Gene expression studies show the anatomical dorsal-ventral gradients along the long-axis of the hippocampus, further supporting an evolutionary basis for dorsal-ventral cellular structure (Amaral and Witter, 1989; Thompson et al., 2008; Strange et al., 2014). This apparent discordance has yet to be resolved, in part due to the methodological and analytic approaches employed.

In addition to the lack of consensus regarding the neurofunctional organization along the longitudinal axis, corroborating evidence has suggested that the left and right hippocampus are distinctly different—with the left hippocampus having more consistent and stable organization than the right hippocampus. However, the morphometric nature of the parcellations has been inconsistent. Important work by Zhong et al. (2019) offers some insight into this. They demonstrated that clustering based on static resting state connectivity resulted in fewer clusters, but that examining dynamic functional connectivity in resting state data resulted in much more granular parcellations, with the left hippocampus clustering into 6 parcellations and the right into 5 parcellations. Furthermore, they demonstrated that state-dependent parcellations changed the clustering of both the left and right hippocampus. Similarly, Plachti et al. (2019) performed a comprehensive clustering of the left and right hippocampus using a multipronged approach. In their work, they used meta-analytically driven clustering as well as resting-state to compare across methodological approaches. While there was some concordance across approaches, differences emerged as well. This highlights two important considerations: (1) parcellations are likely sensitive to task demands (i.e., state-dependency), and (2) analytical choices may alter parcellations. While we do not address the latter in this paper, we do seek to address the issue of task demands.

Parcellation of the hippocampus as a function of task features is not a new concept. Prince et al. (2005) demonstrated strong evidence for an anterior-posterior parcellation that corresponded to an encoding-retrieval gradient. Similarly, Duncan et al. (2014) demonstrated functional connectivity differentiation between encoding and retrieval processes within specific hippocampal subfields, such that functional connectivity between area CA1 and the ventral tegmental area (the primary dopaminergic afferent) predicted long-term memory for associations. Furthermore, functional connectivity between area CA1 and the dentate gyrus/CA3 region was not only indicative of retrieval success, but was also stronger during the retrieval task compared to the encoding task. These results support the fundamental argument that the hippocampus maintains a neurofunctional topographical organization, but it does not address the question of whether other neurocognitive processes utilize these sub-regions, as all of the aforementioned studies used a single paradigm that was parametrically manipulated to examine one specific aspect of memory formation. Most studies of hippocampal sub-specialization limit their investigations to a single behavioral domain or a single paradigm that compares specific neurocognitive processes (i.e., encoding vs. retrieval) (Giovanello et al., 2009; Nadel et al., 2012; Duarte et al., 2014; Nakamura and Sauvage, 2015; Przeździk et al., 2019). This led to a series of studies that used meta-analytic techniques to better characterize the functional and anatomical subfields of the hippocampus (Chase et al., 2015; Persson and Söderlund, 2015; Robinson et al., 2015, 2016; Plachti et al., 2019). While these approaches are robust, they may lack granularity. For example, Hrybouski et al. (2019) examined encoding and retrieval of various types of stimuli. They found that encoding elicited similar activation across all hippocampal subfields, but that retrieval was dependent on the task/stimulus. Meta-analytic approaches may lack the specificity to identify such nuanced differences. Thus, it is important for studies to examine hippocampal functioning using both task and resting state conditions.

To examine hippocampal neurofunctional topography using similar analytical approaches that have been implemented in meta-analytic studies, we employed a hierarchical clustering algorithm to a series of hippocampally engaging tasks collected using ultra high-resolution (i.e., submillimeter), high-field (7T) functional magnetic resonance imaging (fMRI) data. In short, the main idea of hierarchical clustering, as applied to fMRI data, is that voxels that are more related to nearby voxels than to voxels more distant (in terms of Euclidean distance) are subsequently clustered together. Individual voxel timeseries in the left and right hippocampal formation were extracted across a number of tasks designed to engage the hippocampus, as well as during a resting state scan. We believe that this approach could lead to transformative knowledge about the role of the human hippocampus in various neurocognitive processes, while also providing evidence for neural indices of healthy hippocampal function. Understanding the topography of the hippocampus could yield transformative insights into neurocognitive processes, as well as disease pathology.

## Materials and Methods

### Participants

Participants were recruited from the Auburn University community as part of a study examining hippocampal function. The study was approved by the Auburn University Institutional Review Board. All participants provided informed consent. A total of 35 participants were recruited, with usable data from 31 participants (1 participant discontinued because of claustrophobia, 1 participant withdrew due to cramps, 1 participant did not follow instructions, and the scanner experienced technical difficulties for one participant’s visit) (26 right-handed, 12 males, age = 21.13 ± 1.43 years). All participants reported no diagnosed psychological or neurological disorder. This was a within-subject design whereby all participants completed all tasks. Because some participants had unusable data for some scans, we report on only the usable data for each condition. Twenty participants (64.5%) had usable data for all tasks, with an additional 7 (22.6%) having data for 3 of the 4 tasks presented. Thus, there was significant participant overlap for the tasks reported herein. For each task, we report demographics of the subset from the initial 31 participants.

### Functional Magnetic Resonance Imaging

We examined submillimeter fMRI datasets collected at Auburn University on a selection of tasks that had similar neurocognitive constituents to the neurofunctional topography identified in prior meta-analytic studies (Robinson et al., 2015; Plachti et al., 2019), including a face-name paired associate task, and encoding/recognition of words, faces, and scenes. It is important to note that these tasks were not tailored to this analysis, rather they were part of an on-going study examining hippocampal engagement and thus they were not designed for validation of the previously defined meta-analytic models. However, the cognitive processes were similar to the behavioral paradigms associated with some of the hippocampal segments. All tasks utilized the same EPI sequence parameters, which were optimized for the hippocampus, but included most of the brain (37 slices acquired parallel to the AC-PC line, 0.85 mm × 0.85 mm × 1.5 mm voxels, TR/TE: 3,000/28 ms, 70° flip angle, base/phase resolution 234/100, A > P phase encode direction, iPAT GRAPPA acceleration factor = 3, interleaved acquisition). Data were acquired on the Auburn University Siemens 7T MAGNETOM outfitted with a 32-channel head coil by Nova Medical (Wilmington, MA). Stimuli were presented using a back projection Avotec presentation and response system. A whole-brain high-resolution 3D MPRAGE image (256 slices, 0.63 mm × 0.63 mm× 0.60 mm, TR/TE: 2,200/2.8, 7° flip angle, base/phase resolution 384/100%, collected in an ascending fashion) was also acquired for registration purposes.

#### Neurocognitive Tasks

All tasks were performed in a single experimental session. The tasks were presented in the following order: encoding of faces/words/scenes, a task unrelated to the current manuscript, the face-name paired associate task, the resting state scan, and then the recognition of faces/words/scenes task.

##### Face-Name Paired Associate Task

**FIGURE 1:**
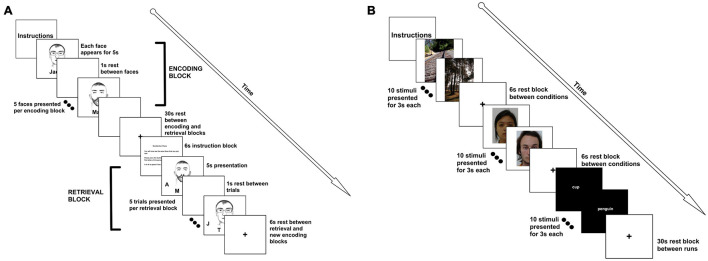
FMRI tasks. **(A)** For the face-name paired associate task, participants were presented with 5 faces. Each face was presented for 5 s with a 1 s interstimulus interval. This constituted an “encoding” block. A 30 s rest period followed the encoding block. Then, participants were given a 6 s instruction slide indicating that they would now need to recall the names of the faces. Participants were then given each of the faces, with initials underneath them. The participant had to select the correct initial using a button box. Faces and distractor initials were randomized. An encoding and retrieval block together constituted a run, and we completed 6 runs for a total of 30 face-name pairings. **(B)** For the encoding and recognition tasks, participants were presented with 10 scenic pictures (each picture was presented for 3 s), followed by a 6 s rest. Participants were then shown 10 faces, with a 6 s rest, followed by 10 words. This constituted a run. There was a 30 s rest between runs. Faces, words, and pictures were the same for all 3 of the runs. The recognition task followed the same format, but was presented at the end of the scan, approximately 45 min later. For the recognition task, participants were asked to push the left button if they had seen the face, word, or picture previously, or the right button if it was novel (40% of the stimuli were novel). For both tasks, clustering was performed using block analyses.

The face-name paired associate task has been one of the most reliable tasks known to elicit hippocampal activation (Tsukiura and Cabeza, 2008; Glahn et al., 2010; Tsukiura et al., 2010). For this task, participants were asked to view a series of faces, each paired with a name (i.e., the “encoding” phase). Following a brief rest period, they were shown a face, and asked to select the name of the face (i.e., the “recognition” phase). Face-name pairings were presented in blocks (6 blocks of encoding/6 blocks of recognition, total acquisition time = 10:06), with 5 pairings per block (stimuli were presented for 5,000 ms, with a 1,000 ms interstimulus interval). Each block was followed by a rest period (30 s). An instruction screen appeared for 6,000 ms prior to the recognition phase (30 s). A rest period followed the recognition phase (as well as an instruction screen about the upcoming encoding block), and a new set of face-name pairings was presented (Figure 1A). During the retrieval phase, participants were asked to respond to which initial at the bottom of the screen corresponded to the face’s associated name via a button box. All participants performed a practice run before entering the scanner. Participants made a decision on a total of 30 face-name pairings, with block analyses performed, rather than individual trial analyses. Data from 22 right-handed participants [14 females/8 males; age (M ± SD) = 21.32 ± 1.49 years] were analyzed.

##### Encoding and Recognition of Faces, Scenes, and Words

For this paradigm, a simple block design was implemented in which participants try to remember 10 faces, 10 words, and 10 visual scenes. Each stimulus was presented for 3,000 ms and participants were asked to view the stimuli, and to try their best to remember the images. Between blocks, there was a 6,000 ms delay, before the next block started. After a full run (1 block of faces, 1 block of words, and 1 block of pictures) was presented, there was a 30 s delay, and then the procedure was repeated for a total of 3 times (Figure 1B). The total task time was 6:09 (6:06 with an extra TR at the end of the scan). In the recognition portion (which was performed at the end of the scanning session, approximately 45 min after the encoding portion of the study), the design was identical, except blocks were mixed with novel and familiar stimuli. Participants were asked to make a decision via button push as to whether or not they had seen the stimulus before. As such, participants had to make a decision for 30 faces, 30 words, and 30 visual scenes. For this task, data from 28 right-handed participants (19 females/9 males; age = 21.27 ± 1.46 years) were analyzed. Words and faces were all of neutral affect.

##### Resting-State

We performed high-resolution resting state fMRI (rs-fMRI) in thirty-one healthy individuals (26 right-handed, 12 males, age = 21.13 ± 1.43 years) using the same EPI sequence described above (100 time points, total acquisition time 5:00). Participants were asked to lay still with their eyes closed, without thinking about anything in particular.

#### Functional Magnetic Resonance Imaging Analysis

Prior to implementing the clustering analyses described below, functional neuroimaging data were preprocessed using FSL neuroimaging analysis software (FMRI Expert Analysis Tool; Smith et al., 2004; Jenkinson et al., 2012; FMRIB, 2021). Specifically, non-brain material was removed from the data, slice timing and motion correction procedures were implemented, and a high-pass temporal filter was applied. Data were smoothed with a 1 mm FWHM Gaussian kernel, to preserve resolution. Functional images were registered to their high-resolution anatomical volume, and standardized to Montreal Neurological Institute (MNI) space.

### Defining Human Hippocampal Region of Interests

We used the Harvard-Oxford Structural Probability Atlas distributed with the FSL neuroimaging analysis software package^1^ (Smith et al., 2004; Jenkinson et al., 2012) to define right and left human hippocampal Region of Interests (ROIs) for inclusion in our analyses. Each ROI was thresholded at 75% probability, yielding a conservative anatomical representation (i.e., hippocampal voxels had to be present in at least 28 of the 37 participants that comprised the atlas). We performed clustering analyses within the hippocampal formation (left and right, separately) to determine neurofunctional segmentations using real data.

### Clustering

Clustering is an unsupervised learning method with a goal of grouping objects into some number of clusters so that objects within a cluster are similar to each other, and objects of different clusters are dissimilar. Here, we implemented hierarchical clustering which does not require *a priori* specification of the number of clusters. Hierarchical clustering has been employed regularly in the analysis of both anatomical and functional MRI data (Cordes et al., 2002; Liao et al., 2008; Wang and Li, 2013; Shams et al., 2015; Siless et al., 2018; Zhao et al., 2018a,b). Here, we applied hierarchical clustering on the entire time series obtained from individual voxels within the hippocampus, independently for task or resting state scans. The entire clustering analysis pipeline was implemented using custom written code in MATLAB.

#### Notation

Let **Z**={**Z**_1_,⋯,**Z**_*j*_,⋯,**Z**_*N*_} represent a set of *N* data objects. In our case, *Z*_*N*_ is the fMRI time series in *N* voxels extracted from the hippocampus. Therefore, *Z* is a matrix containing all voxel time series in the hippocampus. Clustering is eventually performed on Z. Therefore, in order for the clustering to represent a partition at the group level (and not at the individual subject level), we concatenate time series obtained across *M* different subjects and use the concatenated time series in *Z*. In this case, each *Z*_*j*_ in *Z* would be given by **Z**_*j*_=(*Z*_*j*1_,*Z*_*j*2_,⋯,*Z*_*jp*_)∈R_*p*_, where *p* equals the length of the concatenated time series and R is the vector space spanned by ***Z***. Assuming the data is then partitioned into *K* clusters, each cluster is a set of indexes from {1,⋯,*N*}, and each object **Z**_*j*_ belongs to exactly one cluster.

#### Hierarchical Clustering (Agglomerative)

**FIGURE 2:**
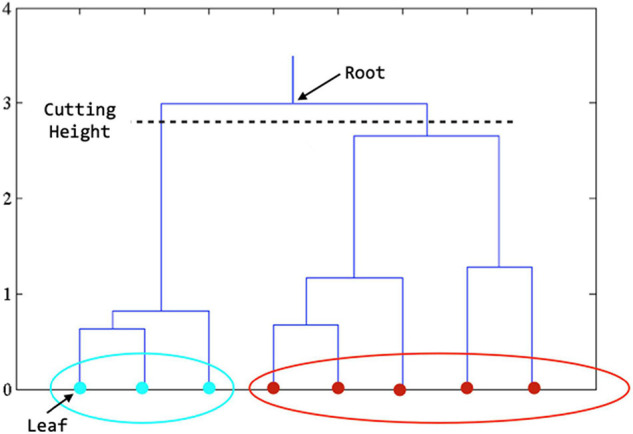
Dendrogram derived from hierarchical clustering. The final clustering result is obtained by cutting the tree at the defined level. In the figure above, when the tree is cut at the level shown, we get the features in teal color in one cluster and those in red color in another cluster.

The main idea of hierarchical clustering (Dasgupta and Long, 2005; Cheng et al., 2006) is that objects that are more related to nearby objects than to objects farther away, in terms of Euclidean distance, are clustered together. A brief description of the procedure is described as follows: (1) assign each object **Z**_*j*_ in a cluster of its own, (2) calculate the distance between any two clusters and merge the closest pair of clusters, and (3) repeat steps 2 and 3 until all **Z**_*j*_ are in one big cluster. The results of hierarchical clustering are usually depicted by a tree-like structure, known as the dendrogram (see Figure 2). The root of the dendrogram represents the entire data, each leaf represents one object, and the height of the dendrogram represents the distance between each pair of clusters. Different data partitions can be obtained by cutting the dendrogram at different levels. Note that the distance between two clusters can be measured in a variety of ways, referred to as linkage methods. The single linkage calculates the shortest distance between two clusters, the complete linkage calculates the longest distance, and the average linkage calculates the average (Andreopoulos et al., 2009). The single linkage can handle non-elliptical shapes of clusters, but can be affected by noise and outliers. The complete linkage is less sensitive to noise and outliers but tends to break large clusters. The average linkage is a compromise between single-linkage and complete-linkage. Thus, the average linkage method was employed in this work.

#### Input Parameter Optimization

Because there are several user-specified input parameters which can significantly affect clustering results, such as the cutting height of the dendrogram, we optimized these parameters using the Calinski-Harabasz (CH) index (Calinski and Harabasz, 1974). Let **C**_*k*_ represent the center of clusters *k*, where 1 ≤ *k* ≤ *K*, and **C** represent the center of entire data set, then the CH index is defined as:


(1) $$C\,H\,\left(K\right)=\frac{B\,\left(K\right)/\left(K-1\right)}{W\,\left(K\right)/\left(N-K\right)}$$

Where the between-cluster variation *B*(*K*) is computed by,


(2) $$B\,\left(K\right)=\sum_{k=1}^{K}{||{\text{C}}_{k}-\text{C}||}^{2}$$

And the within-cluster variation *W*(*K*) is computed by:


(3) $$W\,\left(K\right)=\sum_{k=1}^{K}\sum_{Z_{j}\in c\,l\,u\,s\,t\,e\,r-k}{||{\text{Z}}_{j}-{\text{C}}_{k}||}^{2}$$

Based on the definition of clustering, we want to minimize *W*(*K*) and maximize *B*(*K*). Thus, the optimal parameters are determined by maximizing the CH index. The optimal number of clusters can be identified, simultaneously. Specifically for hierarchical clustering, we started with a relatively high cutting height for the dendrogram. In each iteration, the cutting height was reduced by 1% and the CH index was computed and recorded based on the current data partition. The iteration continued until the cutting height was smaller than a specified baseline (e.g., the average height of the dendrogram). The optimal height was determined as the one with the largest CH index.

#### Visualization of Results

After obtaining clustering results, the identified clusters were mapped back to the image space and overlaid on the anatomical image for visualization of the hippocampal parcellations. Clustering similarities between group and individual results were assessed by using Torres’ method (Torres et al., 2009). Let *C* = {*C*_1_,*C*_2_,⋯,*C*_*m*_} and *D* = {*D*_1_,*D*_2_,⋯,*D*_*n*_} represent two clustering results. The similarity matrix for *C* and *D* is an *m*×*n* matrix defined as:


(4) $$S_{C,D}=\left[\begin{array}{c} \begin{array}{c} S_{11}\;.. \end{array}\\ \begin{array}{c} S_{i\,1}\;..\\ S_{m\,1} \end{array} \end{array}\,\,\begin{array}{c} \begin{array}{c} S_{1\,j}\;..\\ \text{K} \end{array}\\ \begin{array}{c} S_{i\,j}\;..\\ S_{m\,j}\;.. \end{array} \end{array}\,\,\begin{array}{c} \begin{array}{c} S_{1\,n} \end{array}\\ \begin{array}{c} S_{i\,n}\\ S_{m\,n} \end{array} \end{array}\right]$$

Where *S*_*ij*_=*p*/*q*, which is Jaccard’s Similarity Coefficient with *p* being the size of intersection and *q* being the size of the union of cluster sets *C_i_* and *D_j_*. The similarity of clustering *C* and *D* is then defined as:


(5) $$S\,i\,m\,\left(C,D\right)=\frac{\sum_{i\leq m,j\leq n}S_{i\,j}}{\text{max}\,\left(m,n\right)}$$

From Eqs. (4) and (5), it can be seen that 0 ≤ *Sim*(*C*,*D*) ≤ 1, and *Sim*(*C*,*D*) = 1 when two clustering results are identical. Thus, this “similarity index” represents the convergence, or correlation, between the individual’s cluster map to the group map, and may be considered an index of stability.

#### Similarity Indices

We conducted repeated measures analysis of variance (ANOVA) to determine hemispheric and task differences in cluster stability. Because similarity measured how each individual’s cluster profile mapped onto the group-level clustering, a higher similarity index was considered to be a measure of cluster stability, as it represents less inter-subject variance with the group-level cluster solution. For example, if participants, on average, demonstrated a high similarity index, it would indicate that the cluster solution for each individual participant was close to the overall group map. Thus, a high similarity index would indicate that the clusters were stable across participants. We also report the similarity index for hierarchical clustering across all tasks.

## Results

### Similarity Indices

#### Hierarchical Clustering Stability

**FIGURE 3:**
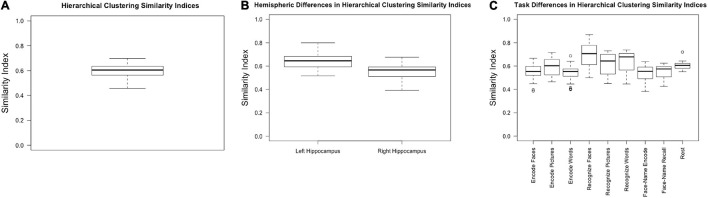
Similarity indices for **(A)** hierarchical clustering across all participants, hemispheres, and tasks; **(B)** hierarchical clustering within the left and right hemispheres, collapsed across task; **(C)** hierarchical clustering within tasks, collapsed across hemisphere.

Collapsing across tasks (including rest), hierarchical clustering resulted in an average similarity index of 0.60, with a standard deviation of 0.06 and a range of 0.46–0.70 (Figure 3A).

#### Hemisphere Stability

We expected the left hippocampus to have higher similarity indices based on previous research which has shown that the right hippocampus has less stable cluster solutions (Robinson et al., 2015; Plachti et al., 2019). Consistent with previous literature, the left hippocampal similarity indices, collapsed across conditions, were higher (0.64 ± 0.07) than the right (0.55 ± 0.07) [*F*_(1,__30__)_ = 50.630, *p* < 0.001, partial η^2^ = 0.628] (Figure 3B).

#### Task Stability

There was a main effect of task [*F*_(__3.387_, _57.575__)_ = 14.286, *p* < 0.001, partial η^2^ = 0.457]. Because sphericity was violated, repeated measures ANOVA results are reported with Greenhouse-Geisser epsilon, the most conservative adjustment to the degrees of freedom. *Post-hoc* pairwise comparisons (Bonferroni corrected) indicated that encoding faces (0.53 ± 0.08) had a lower similarity index compared to recognizing faces (0.67 ± 0.11, *p* = 0.001), pictures (0.60 ± 0.10, *p* = 0.029), and words (0.63 ± 0.10, *p* = 0.004). Encoding pictures (0.59 ± 0.08) had a lower index compared to recognizing faces (*p* = 0.003). Encoding words (0.53 ± 0.068) had a significantly lower index than recognizing faces (*p* < 0.001), pictures (*p* = 0.042), and words (*p* = 0.006). Face recognition had a significantly higher similarity index compared to all other task conditions (*p* ≤ 0.040 for all conditions) but was not significantly different from rest (*p* = 0.471). Word recognition had a significantly higher similarity index compared to both face-name paired associate tasks (encoding: 0.53 ± 0.08, *p* = 0.013; recall: 0.55 ± 0.06, *p* = 0.021) (Figure 3C and Table 1).

**TABLE 1:** Similarity index descriptive statistics, by task, condition, and hemisphere.

**Similarity index descriptive statistics**						

**Task**	**Hemisphere**	**n**	**Minimum**	**Maximum**	**Mean**	**SD**
Encode faces	Left	27	0.33	0.74	0.59	0.09
Encode faces	Right	27	0.25	0.60	0.50	0.08
Encode pictures	Left	27	0.50	0.91	0.70	0.14
Encode pictures	Right	27	0.35	0.58	0.49	0.06
Encode words	Left	27	0.40	0.81	0.59	0.09
Encode words	Right	27	0.25	0.59	0.48	0.08
Face-name Paired associate encode	Left	21	0.25	0.63	0.53	0.09
Face-name paired associate encode	Right	21	0.33	0.66	0.54	0.09
Face-name paired associate recall	Left	21	0.42	0.68	0.55	0.06
Face-name paired associate recall	Right	21	0.33	0.68	0.55	0.09
Recognize faces	Left	25	0.50	0.91	0.72	0.14
Recognize faces	Right	25	0.50	0.86	0.66	0.13
Recognize pictures	Left	25	0.50	0.91	0.73	0.13
Recognize pictures	Right	25	0.29	0.62	0.50	0.09
Recognize words	Left	25	0.50	0.88	0.73	0.12
Recognize words	Right	25	0.34	0.67	0.55	0.10
Resting state	Left	31	0.55	0.81	0.63	0.05
Resting state	Right	31	0.49	0.67	0.59	0.04
Valid n (listwise)		18				

*Some task data for individual participants did not yield stable cluster solutions, and are not included in the similarity indices (n = 1 for encoding, n = 1 for face-name paired associates task, n = 3 for recognition).*

### Cluster Results

All hierarchical clustering maps are available via https://github.com/jennyrobinson/hipp-neurofunc-clusters.git.

#### Tasks vs. Rest

**FIGURE 4:**
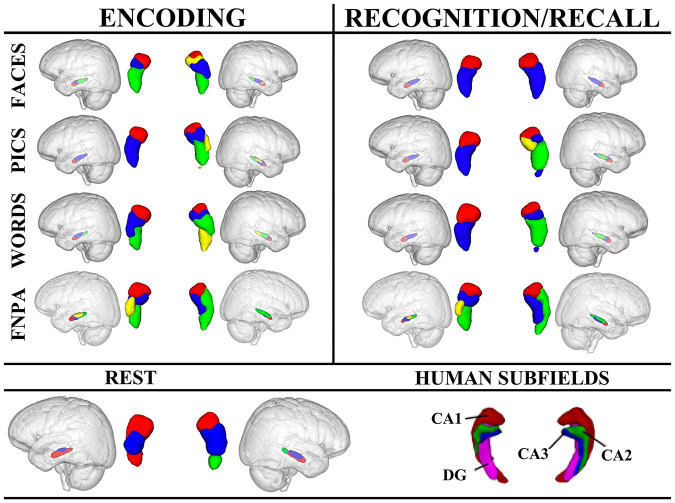
Hierarchical clustering results for left and right hippocampi across tasks and conditions. Human hippocampus subfields are also presented for reference. For encoding, the left anterior hippocampal cluster, depicted in red, appears to align with CA1, while in the right hippocampus, the anterior-most cluster may also encompass portions of CA2 (yellow) in addition to CA1. Clustering in the right hippocampus during the FNPA task appears to alright with CA1 (red), and CA1/CA2 (green) and CA3/DG (blue). FNPA, face-name paired associate task; DG, dentate gyrus.

Across all tasks, functional parcellations were largely along the longitudinal axis with some medial-lateral configurations, consistent with recent investigations (Chase et al., 2015; Robinson et al., 2015, 2016; Zhong et al., 2019; Cheng et al., 2020). However, resting-state functional parcellations were oriented more along the short-axis (dorsal-ventral), similar to the cytoarchitectonic atlases (Amunts et al., 2005; Eickhoff et al., 2007; Plachti et al., 2019). Regardless of whether clustering was performed on task or non-task data, the left parcellations were consistently fewer and more stable compared to the right, as noted by the similarity indices (Figure 4).

#### Encoding

Across conditions, encoding appeared to be affected by stimulus type. For example, face encoding resulted in 3 clusters in the left hippocampus and 4 in the right, whereas picture encoding only resulted in 2 clusters in the left and 4 in the right. Encoding words had a similar clustering to encoding faces. Face-name paired associate coding, which requires components of both word and face encoding, plus the association of the information, elicited a different morphometric clustering with the left hippocampus splitting into 4 clusters, and the right into 3. Interestingly, the encoding and recall of face-name information had nearly identical clustering. These data point toward different clustering profiles as a result of both stimulus type and process (simple encoding vs. paired-associations) (Figure 4).

#### Recognition and Recall

Recognition in the left hippocampus was nearly identical for faces, pictures, and words, each with high similarity indices suggesting that cluster results were consistent across participants. However, the right hippocampus exhibited different clustering morphometries depending on the stimulus, with faces having a simple 2 cluster solution with a high similarity index compared to recognition of pictures or words which had 4 and 3 cluster solutions, respectively. Additionally, face-name paired associate recall exhibited a medial-lateral cluster orientation in comparison to the recognition tasks (Figure 4).

## Discussion

Advancing our understanding of the neurofunctional characteristics of the hippocampus could have a transformative impact in neuroscience as well as psychiatry and neurology. Mounting evidence suggests that different hippocampal subfields may be implicated in the etiology of certain neurological and psychiatric conditions (Huang et al., 2013; Haukvik et al., 2015; Treadway et al., in press). In this study, we present evidence for task-specific influences on intrahippocampal clustering. Additionally, we characterize unique morphometric clusters between resting state and task conditions. Our findings suggest that these functional clusters also differ as a consequence of task phase, stimulus type, stimulus features, state, and hemispheric asymmetries, highlighting the dynamic functional characteristics of the hippocampus.

Examining the neurofunctional profiles obtained from the hierarchical clustering analysis, we observed an interesting pattern—during encoding, stimulus context appeared to have important implications for intrahippocampal clustering (i.e., clustering profiles were different depending on whether the participant was encoding a face vs. encoding a word). However, during recognition, the left hippocampus remained largely the same, regardless of whether there was a face, picture, or word. Conversely, Hrybouski et al. (2019) observed a different pattern; in their study, encoding (regardless of memory type) equally activated all subregions of the hippocampus, while a posterior to anterior gradient in activation was only observed across subfields for all types of memory during retrieval. However, Hrybouski et al. (2019) utilized the “designs” subtest of the Wechsler Memory scale, in which participants must recall the shape of a grid of abstract symbols. In the present study, participants viewed faces, pictures, and words, which represents a more ecologically valid stimulus set. There may be a familiarity effect reflected in differential activation, particularly during the encoding phase, that is unique to memory tasks with more familiar or common stimuli. Alternatively, differences may reflect differences in the application of the clustering methods. For example, here we examined within-hippocampus connectivity patterns as opposed to clustering based on each voxels connectivity to the rest of the brain. These differences in activation patterns highlight the need to study the neurofunctional properties of the hippocampus using a wide variety of stimulus presentations, and using both intrahippocampal connectivity patterns as well as whole-brain connectivity patterns.

These data provide preliminary support for topographical organization, but they also highlight the need for refinements that take into account more than stimulus features. For example, in examining encoding of faces vs. encoding of pictures in a traditional general linear modeling analyses, we saw different patterns of activation (not shown here), providing motivation for exploring task parameters for which the hippocampus may have preferential responding (i.e., it is possible that there is a stimulus type × process interaction, necessitating the study of these constructs independently). Furthermore, other evidence suggests that inter-individual variation in hippocampal organization predicts recollection ability better than characterization based on functional parcellation. Individual differences in hippocampal organization are related to success and failure in memory encoding tasks, suggesting an important need to emphasize individual differences in hippocampal studies. It also lends further support to evidence positing a functional significance to the gradient-based organization of the hippocampus as well (Przeździk et al., 2019).

Additionally, very few studies address the issue of lateralization when discussing the long-axis anterior-posterior gradient, despite strong evolutionary and functional neuroimaging evidence suggesting that the right and left hippocampus are likely to have functional differences (Gagliardo et al., 2005; Klur et al., 2009; Churchwell and Kesner, 2011; Persson et al., 2013; Hami et al., 2014). For example, Klur et al. (2009) noted lateralization differences in the rat via gene expression profiling and reversible inactivation (via lidocaine), such that the left hippocampus demonstrated preferences toward information transfer, and the right hippocampus favored spatial navigation storage and retrieval processes. Similarly, Freeman et al. (2004) identified hemispheric differences in non-human primates. These lateralization differences appear to be preserved (i.e., dissociable roles of the hippocampus have been hypothesized across species; Ekstrom et al., 2003; Freeman et al., 2004; Gagliardo et al., 2005; Klur et al., 2009; Persson et al., 2013; Copara et al., 2014; Hami et al., 2014; Herold et al., 2014; Jonckers et al., 2015), and have been linked to gender differences in humans (Persson et al., 2013). Most have theorized that the right hippocampus is primarily engaged in spatial processing (i.e., 3D spatial navigation, or remembering an object location), while the left is more attuned to verbal information (Greve et al., 2011; Duarte et al., 2014). This phylogenetic preservation suggests that the topography of the hippocampus serves an important functional role and elucidating the geography may inform the development of transformative computational models of how the brain works under hippocampal-dependent cognitive and emotive processes. This investigation observed high similarity in clustering solutions in the left hippocampus during recognition and recall for faces, pictures, and words, while faces had a 2-cluster solution in the right hippocampus and pictures and words had 4 and 3 cluster solutions, respectively. However, during encoding, 3 clusters were observed in the left hippocampus and 4 in the right hippocampus for faces but picture encoding only resulted in 2 clusters in the left and 4 in the right. These results indicate that stimulus type and task phase may interact with hemispheric asymmetries in the neurofunctional characteristics of the hippocampus.

Recent work by Zhong et al. (2019) examining dynamic hippocampal-cortical functional connectivity provided evidence for state-dependent functional segmentation of the hippocampus. Our work corroborates the notion that state matters and extends it by also providing evidence that stimulus features may also impact clustering profiles. With respect to state-dependent functional clustering of the hippocampus, our data indicate that resting state aligns well with dorsal/ventral orientation of functional parcellations—in line with the well-known cytoarchitectonic organization. On the other hand, we find that certain features of the stimulus/task used tend to produce clustering profiles along the medial-lateral orientation, which is also likely at least partially influenced by anatomical considerations, given that the lateral segments of the hippocampus can be attributed to CA1-3 and the more medial portions to the subiculum (Plachti et al., 2019; Kharabian Masouleh et al., 2020). Importantly, our work examined intrahippocampal connectivity patterns, which offers new insight into the neurofunctional organization of the hippocampus and can serves to complement what is known about hippocampal-cortical clustering.

This research highlights the need for several lines of future inquiry. First, these results should be replicated on larger samples. Second, we examined static clustering profiles given the length of our tasks, but additional research should examine dynamic functional connectivity which has been shown to parcellate the left and right hippocampus differently than its static counterpart (Zhong et al., 2019; Deshpande and Jia, 2020). This would require longer tasks that would be adequately powered to examine dynamic shifts in connectivity profiles. This would also require that more trials be conducted for each task to accurately delineate the contributions of process and stimulus context. Additionally, behavioral/memory performance should be accounted for and used in future analyses to determine whether functional patterns associated with successful encoding/retrieval parcellate differently. Finally, future studies should parametrically manipulate both cognitive process and affective state as well as stimulus type to better characterize and define the neurofunctional topography of the left and right hippocampus, as these appear to be important factors. Doing so may allow us to better understand hippocampal dysfunction in neurological and psychiatric disorders.

Here, we present a possible theoretical resolution to the discordance between functional and anatomical hippocampal subfields, especially given that anatomically, the hippocampus has withstood evolutionary pressures. Specifically, we suggest that cognitive processing and stimulus features may influence how the hippocampus activates, with non-specific processing (i.e., resting state) eliciting more dorsal-ventral clustering, and task-dependent processing segmenting along the longitudinal and medial-lateral axes as the hippocampus “shifts” to specific processing demands. Furthermore, we highlight the importance of considering neurofunctional clustering using intrahippocampal connectivity patterns, afforded by the improved resolution possible at higher field strengths.

## Data Availability Statement

The datasets presented in this article are not readily available because this would jeopardize patient/participant privacy. The anonymized data is available pending approval from the Auburn University Institutional Review Board. Requests to access the datasets should be directed to corresponding author.

## Ethics Statement

The studies involving human participants were reviewed and approved by the Auburn University Institutional Review Board. The patients/participants provided their written informed consent to participate in this study.

## Author Contributions

JR conceptualized the project, created tasks, acquired the data, and wrote the first version of the manuscript. XZ and GD performed the clustering analysis. SB, RB, ML, GD, and SN contributed to the theory and development of the manuscript. All authors contributed to the article and approved the submitted version.

## Conflict of Interest

XZ was employed by Quora, Inc. JR has a paid consultant relationship with VDF FutureCeuticals, Inc. Neither Quora, Inc. or VDF FutureCeuticals were involved in any aspect of this project. The remaining authors declare that the research was conducted in the absence of any commercial or financial relationships that could be construed as a potential conflict of interest.

## Publisher’s Note

All claims expressed in this article are solely those of the authors and do not necessarily represent those of their affiliated organizations, or those of the publisher, the editors and the reviewers. Any product that may be evaluated in this article, or claim that may be made by its manufacturer, is not guaranteed or endorsed by the publisher.

## Footnotes

1
http://www.fmrib.ox.ac.uk/fsl/fslview/atlas-descriptions.html#ho
